# Intracardiac biopsy of cardiac tumors with echocardiographic guidance: Case report

**DOI:** 10.3389/fcvm.2023.1103918

**Published:** 2023-04-27

**Authors:** Jinyun Zhu, Ning Zhang, Qunchao Ma, Luhang Jin, Xiaohong Pan

**Affiliations:** ^1^ Department of Cardiology, Second Affiliated Hospital, College of Medicine, Zhejiang University, Hangzhou, China; ^2^ Department of Cardiology, the Affiliated Hangzhou First People’s Hospital, College of Medicine, Zhejiang University, Hangzhou, China

**Keywords:** intracardiac echocardiography, endomyocardial biopsy, primary cardiac tumor, case reports

## Abstract

**Background:**

Primary cardiac tumors are very rare, and about 20–30% of them are malignant tumors**.** Since early signs of cardiac tumors are non-specific, diagnosis can be challenging. There is a lack of the recommended guidelines or standardized strategies for diagnosis and optimal treatment for this disease. As the definite diagnoses of most tumors are made by pathologic confirmation, biopsied tissue is essential in determining the treatment for patients with cardiac tumors. Recently, intracardiac echocardiography (ICE) has been introduced to assist biopsy procedures of cardiac tumors and it provides high-quality imaging.

**Case Description:**

Due to its low prevalence and variable presentation, cardiac malignant tumors usually are easily missed. Hereby, we report three cases of patients who presented with non-specific signs of cardiac disorder and was initially suspended diagnosis as lung infection or cancer. Under the guidance of ICE, cardiac biopsies were successfully on cardiac masses, giving critical data for diagnosis and treatment planning. No procedural complications were obtained in our cases. These cases are intended to highlight the clinical value and importance of ICE-guided biopsy of intracardiac mass.

**Conclusions:**

The diagnosis of primary cardiac tumors relies on the histopathological results. In our experience, using ICE for biopsy of an intracardiac mass is an attractive tool to increase diagnostic results and reduce the risk of cardiac complications associated with inadequate targeting of the biopsy catheters.

## Introduction

Primary cardiac tumors are extremely uncommon, and the majority of them are benign. It was reported that the prevalence of primary cardiac tumors is between 0.001 to 0.28% in autopsy series (1). About 40% of malignant tumors are angiosarcomas, which make up the majority of sarcomas. Cardiac angiosarcomas are a rare group of soft tissue sarcomas, characterized by aggressive local growth and early spread. The majority develop in the right atrium, and can inﬁltrate into neighboring structures and spread distantly (2). Another uncommon hemangioma that develops from pre-endothelial or vascular endothelial cells is cardiac epithelioid hemangioendothelioma (EHE). Its biological behavior is between benign hemangioma and malignant angiosarcoma, and it has local invasiveness and metastatic potential (3). It is difficult to diagnosis due to the initial nonspecific symptoms.

Multimodality imaging and atrial biopsy of a cardiac mass should be performed early as a diagnostic approach (4). Although cardiac biopsy is not a common procedure, tissue diagnosis is crucial for the treatment strategy because the prognosis of cardiac tumors varies greatly depending on the underlying disease. In a patient suspected of having a malignant cardiac tumor who does not have access to curative surgical treatment, histopathological confirmation is a necessary option next to treatment approach. Several methods have been used to replace thoracotomy such as transvenous biopsy, ultrasound-guided transesophageal biopsy and transthoracic biopsy guided by ultrasonography or CT. Intracardiac echocardiography (ICE) is ideally suited to imaging structures in the right heart (5). Although it needs local anesthesia and venous puncture with relatively large catheter, it can provide us a good guidance during the biopsy of cardiac tumors.

Until now, only a few case studies have demonstrated the use of ICE in directing biopsy of cardiac tumor (6–8). Here, we present three cases of right atrium masses treated by biopsy of cardiac masses under ICE guidance. Our cases demonstrated the difficulty in diagnosing cardiac masses due to initial nonspecific symptoms such as dyspnea and shortness of breath. They also highlighted the importance of multimodality imaging and ICE guided atrial biopsy in the early diagnosis of rare cardiac tumors. We hope to provide practical evidence for the use of ICE guided biopsy in the diagnosis of suspected intracardiac mass.

## Case presentation

We herein show three cases of ICE-guidance for biopsy of cardiac masses being used to diagnose cardiac tumor. This study was approved by our institutional review board.

## Case 1

**Figure 1:**
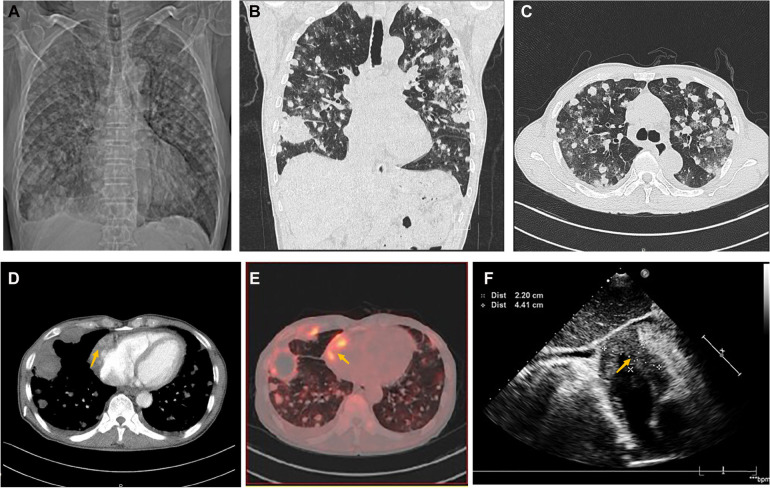
Chest computed tomography demonstrated cardiac mass of Case1. (**A**) Chest x-ray and Chest CT images in axial (**B**) and coronal planes (**C**) showed remarkable extensive pulmonary lesions. (**D**) Contrast-enhanced CT showed a hypodense oval filling contrast defect (arrow) at the right atrium (RA). (**E**) 18F-fluorodeoxyglucose positron emission tomography/CT (18F-FDG PET/CT) showed more uptake in the RA mass (arrow), lung, and pleura. (**F**) Echocardiography depicted an oval-shaped mass (22 × 41 mm) (arrow) in the RA at the level of the superior vena cava ostium.

**Figure 2:**
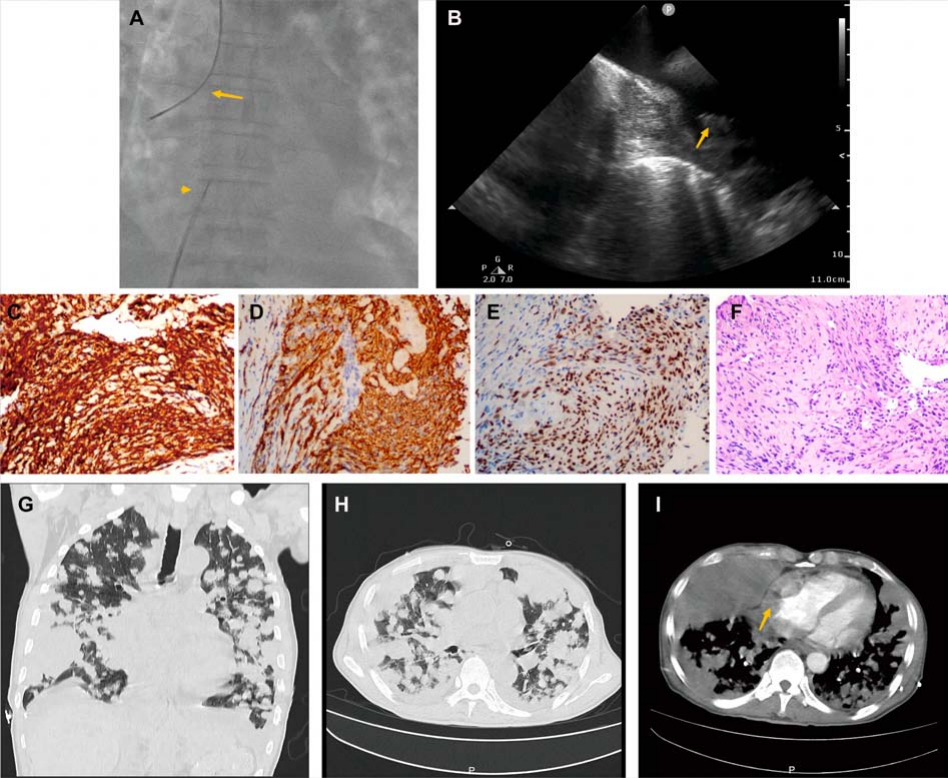
Histological endomyocardial biopsy specimen of the right atrium and lung rapid deterioration of Case1. (**A**) Catheter for intracardiac echocardiography (arrowhead) and cardiac bioptome (arrow). (**B**) The intracardial echocardiography demonstrates the mass of the right atrium and well-targeted biopsy catheter (arrow) on the mass. (**C–F**) The representative histologic aspect of the mass. Immunohistochemical staining for CD31(**C**), CD34 (**D**), and ERG (**E**) marker in cytoplasmic. (**F**) Cells displaying epithelioid morphology, large nuclei, and ample cytoplasm (hematoxylin and eosin). (Original magnification 40X). (**G,H**) Axial and coronal contrast-enhanced CT scans showed rapid progression of lung metastases, and (**I**) recurrent bloody pleural effusion one month after hospitalization. The arrow indicates progressive RA mass.

A 70-year-old man presented with progressive dyspnea and weakness for one month. His physical examination, including the cardiovascular examination, was unremarkable. He had low-grade pyrexia of 37.8°C, with no cough and sputum. Oxygen saturations were 97% on room air. Blood tests showed normocytic anemia (90 g/L), elevated C-reactive protein (CRP) (33.4 mg/L), leucocyte count (12.7 × 10^9^/L), pro-Brain natriuretic peptide level 406 pg/ml, D-dimer of 9840 ug/L, and tumor marker CA125 (312.1 U/ml). Chest X-ray (Figure 1A) and computed tomography (Figures 1B,C) revealed striking extensive pulmonary lesions, a large soft tissue mass in the right atrium (RA), and pleural effusion. He had persistent bilateral bloody pleural fluid. Thoracentesis removed bloody exudative fluid without malignant cells. Superficial lymph nodes ultrasonography revealed left subclavian lymphadenopathy. Pathological results of lung biopsy and lymph nodes were nondiagnostic. Contrast-enhanced CT showed a hypodense oval filling contrast defect at the right atrium (RA) (Figure 1D) 18F-fluorodeoxyglucose positron emission tomography/CT revealed focal uptake in the RA mass (with SUVmax of 5.8), and scattered uptake in lung and pleura metastatic (Figure 1E). Echocardiogram showed a large mass (22 × 41 mm) in the RA invading the superior vena cava (Figure 1F). Ultrasonographical guided right atrial biopsy was performed. An 11F sheath containing an intracardiac ultrasonography catheter (Biosense Webster, Johnson & Johnson, USA) was inserted into the right atrium through the right femoral vein while under local anesthesia with 1% lidocaine. By crossing the tricuspid annulus, the mass' size and extent could be clearly seen. Under the guidance of ICE, a cardiac biopsy forceps (Argon, USA) was inserted into the right atrium *via* the right internal jugular vein and clamped atrial tissue. The RA mass was visualized and five biopsies were performed (Figure 2A,B, Supplementary Appendix Video S1-2). Of those samples, 3 were mixtures of necrotic myocardium, and 2 were diagnostic for EHE. Histopathology results (Figures 2C–F) revealed a spindle cell tumor, staining with CD34, CD31, and ERG expression, confirming the diagnosis of cardiac epithelioid hemangioendothelioma (EHE). A multidisciplinary team (oncologists, cardiologists, and respiratory doctors) evaluated this patient and recommended chemotherapy and targeted therapy with no mass resection. Unfortunately, the patient's condition deteriorated quickly due to aggressive pulmonary lesions (Figures 2G–I), and he had severe dyspnea. The patient made the decision to receive hospice care.

## Case 2

**Figure 3:**
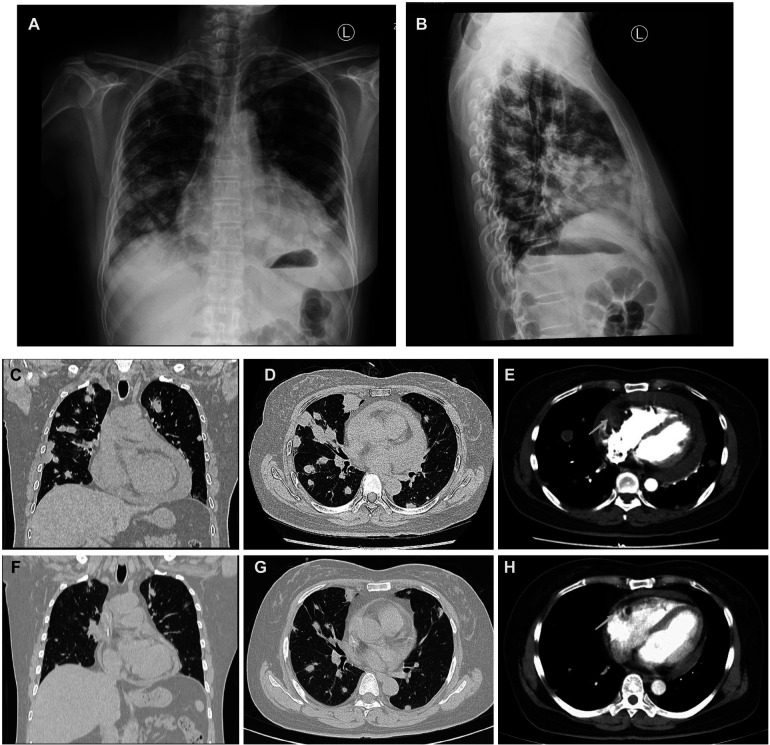
Comparison of lung metastases and cardiac mass before and after chemotherapy of Case2. (**A,B**) Frontal and lateral Chest x-ray demonstrated extensive pulmonary lesions. (**C**) Chest CT at coronal view and (**D**) axial view revealed severe lung metastases. (**E**) Contrast-enhanced Chest CT reveals a mass lesion (arrow) along the superior and posterior right atrial wall without enhancement. After 5 courses of chemotherapy, the number of lung metastases (**F,G**) was less than before (**C,D**), and the lateral wall of the RA (**H**) has no occupied (arrow) compared to earlier (**E**).

**Figure 4:**
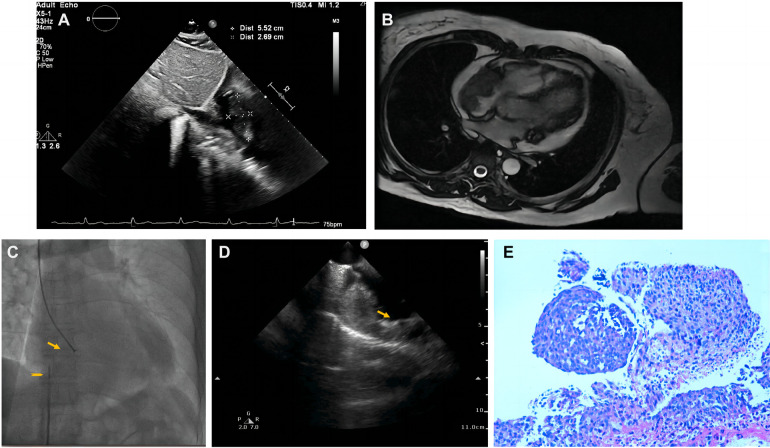
Multimodality images and intracardiac echocardiography-guided biopsy of cardiac tumors of Case2. (**A**) Transthoracic echocardiography showed a huge mass lesion in the RA. (**B**) Cardiac enhanced-MRI revealed a large irregular mass (55.2 × 38.1 × 33.9 mm) with a rounded appearance in the RA and connected with the right atrium. (**C**) Catheter for intracardiac echocardiography (arrowhead) and cardiac bioptome(arrow). (**D**) The intracardial echocardiography demonstrates thickness mass of the right atrium and well-targeted biopsy catheter (arrow) on the mass. (**E**) Histopathological diagnosis revealed cardiac angiosarcoma, with spindle tumor cells.

A 50-year-old woman, who had been experiencing shortness of breath and chest tightness for four months, was admitted to a nearby hospital. A chest computed tomography (CT) revealed enlarged heart with pericardial effusion as well as multiple lesions in lungs with suspected infection. CT-guided needle biopsy of pulmonary lesions was performed. Only heterozygous cells were found in the necrosis, and the pathological findings of the lung biopsy were unremarkable. After a few days, the patient gradually felt dyspnea and weakness. She was transferred to our cardiology department for a conclusive diagnosis. Blood testing showed high level of the C-reactive protein (CRP) (16.6 mg/L), pro-Brain natriuretic peptide level of 224 pg/mL, D-dimer of 2050 ug/L, and tumor marker CA125 (60.1 U/ml). Her chest x-ray (Figures 3A,B) and chest CT (Figures 3C,D) revealed multiple lung lesions. Enhanced chest CT showed an oval hypodense contrast filling defect in the right atrium wall. The size is about 55mm × 28mm × 36 mm (Figure 3E). Transthoracic echocardiography (Figure 4A) and contrast-enhanced Cardiac magnetic resonance (CMR) imaging (Figure 4B) confirmed a large irregular mass (55.2 × 38.1 × 33.9 mm) in the right atrium (RA) invading the superior vena cava. We suspected that this was a cardiac malignant tumor with extensive lung metastases, and then performed an ICE-guided right atrial biopsy (Figures 4C,D, Supplementary Appendix Videos S3-4). Histopathological results (Figure 4E) confirmed the diagnosis of cardiac angiosarcoma, with spindle tumor cells staining positively for CD34, CD31, Fli-1, and ERG, with ki-67 proliferation index reached 60%. After being transferred to the oncology unit, this patient was advised to undergo chemotherapy and targeted therapy without having a mass removed. The patient was treated with chemotherapy (Paclitaxel 207 mg/m^2^ day1, liposomal doxorubicin 30 mg/m^2^ day1, and Anlotinib day1–14 for targeted therapy). After 5 courses of chemotherapy, the lateral wall of the right atrium has no occupied, and the number of lung metastases was less than before (Figures 3F–H).

## Case3

A 62-year-old man was transferred from local hospital to our cardiology department for chest tightness and recurrent pericardial effusion for two months. More than two months ago, the patient experienced chest tightness. When the patient went to the local hospital's emergency room, chest CT showed enlarged cardiac shadow and massive pericardial effusion with interstitial pulmonary edema in the lower lobe of right lung.

**Figure 5:**
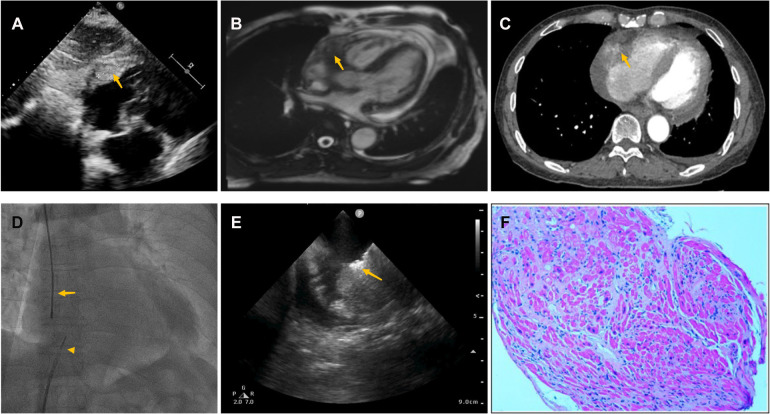
Multiple investigations for diagnosis of Case3. (**A**) Transthoracic echocardiography showed cardiac mass lesion (arrow) in the RA. (**B**) Chest enhanced CT, and (**C**) Cardiac enhanced-MRI revealed a large mass (arrow) at the junction of the RA and RV. (**D**) Catheter for intracardiac echocardiography (arrowhead) and cardiac bioptome (arrow). (**E**) The intracardial echocardiography demonstrates well-targeted biopsy catheter (arrow) on the mass. (**F**) Histopathological imaging revealed small vascular lumen formed by single cell, and cardiac epithelioid hemangioendothelioma (EHE) was diagnosed.

Pericardiocentesis was used to treat his bloody pericardial effusion and drain it. In order to treat the symptoms, diuretics and antihypertensives were given. After one month, the patient once more experienced a significant increase in pericardial effusion along with obvious chest tightness. The patient then went to the emergency department for pericardial effusion drainage. Unexpectedly, his chest tightness and dyspnea immediately got worse. He was admitted to our hospital for further diagnosis. A second pericardiocentesis was carried out, producing 900 ml of bloody fluid in total. Blood tests performed shortly after admission revealed high levels of the C-reactive protein (CRP) (25.4 mg/L), pro-Brain natriuretic peptide (660 pg/ml), D-dimer (3,560 ug/L), and tumor marker CA125 (87 U/ml). Echocardiography showed a hyperechoic mass at the right ventricle lateral wall junction (Figure 5A). Enhanced-CMR and chest CT clearly confirmed that a mass (38mm × 23 mm) was located at the junction of the right atrium and ventricle and had a modest amount of pericardial effusion (Figures 5B,C). Pathological analysis of the pericardial effusion revealed sporadic mesothelial cells and inflammatory cells but no tumor cells. For further diagnosis, we carried out an atrial biopsy under the guidance of the ICE (Supplementary Appendix Videos S5–6). The biopsy technique was the same as above without complications (Figure 5D,E). This result revealed small vascular lumen formed by single cell (Figure 5F). and considered as an intermediate or low-grade malignant vascular tumor with positive CD34, CD31, Fli-1, and ERG expression, confirming the diagnosis of cardiac epithelioid hemangioendothelioma (EHE). With the pericardial effusion's rapid progression and significant dyspnea, the patient eventually discontinued getting treatment.

All procedures performed in this study were in accordance with the ethical standards of the institutional and/or national research committee(s) and with the Helsinki Declaration (as revised in 2013). Written informed consent was obtained from the patient for publication of these case reports and accompanying images.

## Discussion

Primary cardiac tumors are rare, with incidences ranging from 0.001% to 0.030% at autopsy series (9). Among them only 20% are malignant. The malignant primary cardiac tumors are extremely uncommon, whereas metastatic cardiac tumors occur more frequently. Due to the nonspecific nature of the initial symptoms, diagnosis is frequently challenging and delayed (10).

Cardiac angiosarcoma is an uncommon subset of soft tissue sarcomas, and because of its aggressiveness, high rates of local recurrence, and systemic metastases, it is linked to a poor prognosis (11). Because the earliest symptoms of cardiac angiosarcoma are vague and associated with massive pulmonary metastases, our cases illustrated how challenging it is to diagnose this type of cardiac tumor. It implies in the prognosis that more than 50% of these individuals have metastases at the time of diagnosis (12). There is presently no established treatment strategy for this rare condition. When localized, surgery appears to lead to the best outcomes, but this can be technically challenging and not always feasible. Furthermore, some individuals may not be candidates for surgery due to the disease's rapid progression. Cardiac epithelioid hemangioendothelioma (EHE) is a malignant vascular neoplasm, characterized by significant heterogeneity in both clinical presentation and prognosis. Its biological behavior is between benign hemangioma and malignant angiosarcoma, and it has local invasiveness and metastatic potential (13). Metastases and mortality can occur in about 25% and 15% of EHE, respectively (14). Because of its low prevalence and inconsistent presentation, cardiac EHE is frequently overlook.

However, the conclusive diagnosis and gold standard are cytology and immunohistochemistry. Open heart surgery, mediastinoscopy, and metastatic mass exploration are more invasive methods that require general anesthesia. Percutaneous transvenous biopsy of cardiac tumor can be done under the guidance of transesophageal echocardiography (TEE). But for this treatment, general anesthesia is also required. Given these tumors are often located in higher risk areas for perforation (atrial or right ventricular free wall), fluoroscopy alone is insufficient for bioptome guidance, and an additional imaging modality is required to guide the procedure. The use of intracardiac echocardiography (ICE) in cardiac procedures has continuously increased since it was first brought into clinical practice many years ago (15).

Currently, ICE is used for more than just electrophysiologic procedures, transcatheter aortic valve insertion, and percutaneous device closures (16). The operator can adjust the catheter for the position, orientation and angle to acquire the optimal views. Without the use of general anesthesia, ICE can give us clearer cardiac imaging, and it is more practical and patient-friendly (17). ICE can reduce fluoroscopy times, resulting in less radiation exposure and procedural duration. The ICE differs from a CT and an MRI since it uses real-time imaging while being performed.

In this report, we showed the feasibility of the ICE during targeting of the biopsy catheter, and confirmed that use of ICE guided diagnostic myocardial biopsy is safe and accurate for tissue localization. The patients underwent cardiac biopsies without any complications. It can be used for the biopsy of the mass from right-side of the heart, especially in patients with high risk for anesthesia. The experiences for the left heart biopsy will be improved in the future when this approach is combined with trans-septal puncture and intracoronary sinus ICE (18). The conventional chemotherapeutic drugs paclitaxel, docetaxel, and doxorubicin are still in use today. Additionally, angiogenesis inhibitors, such as bevacizumab, sunitinib, and sorafenib, have been used to prevent the proliferation of endothelial cells. There are some studies showed many of these metastatic patients die within a few months after diagnosis (19, 20). Therefore, we must understand how crucial early diagnosis is for cardiac tumors.

However, there have been several technical challenges or limitations in the cardiac biopsy. The fluoroscopy provides very limited information in spatial information. As a result, ensuring precise location and depth in sample acquisition is insufficient. Image temporal resolution is also required because heart beats, respiration and blood flow can all affect target movement. As the wall thickness varies, the operator should be cautious of perforation (especially in left atrium). It is currently used less frequently in left atrial masses and can be combined with other more refined techniques.

## Conclusions

In our cases, heart tumors present with the patient's initial nonspecific symptoms. Multiple investigations should be carried out early on in these patients, and diagnoses must be systematically taken into account. Additionally, ICE-guided atrial biopsies are a reliable and efficient diagnostic technique.

## Data Availability

The original contributions presented in the study are included in the article/**Supplementary Material**, further inquiries can be directed to the corresponding author/s.
