# Supplementary material for: Intracardiac biopsy of cardiac tumors with echocardiographic guidance: Case report
Source: Front Cardiovasc Med. 2023 Apr 27;10:1103918. doi: 10.3389/fcvm.2023.1103918 (PMC10173306; doi:10.3389/fcvm.2023.1103918)
Supplement: Supplementary file 1 [file Table1.docx]

**Table1. Summary of the characteristics of our case series**

| Case series | Gender | Age | Chief  Complaint | Lung metastases | Cardiac  mass | Pericardial effusion | Pathological biopsy | Outcome |
| --- | --- | --- | --- | --- | --- | --- | --- | --- |
| 1 | M | 70 | Dyspnea/weakness  for one month | multiple lung lesions | RA | bloody pleural fluid | Cardiac EHE | PS 4 Grade/get hospice care |
| 2 | F | 50 | chest tightness/  shortness of breath  for four months | multiple lung lesions | RA invading the superior vena cava | pericardial effusion | Cardiac angiosarcoma | 5 courses of chemotherapy |
| 3 | M | 62 | chest tightness for two months | NO | junction of the RA and RV | recurrent bloody pericardial effusion | Cardiac EHE | discontinued  getting treatment. |

*M: Male; F: Female; EHE: Cardiac epithelioid hemangioendothelioma; RA: right atrium; RV: ventricle; PS: Patient*performance status*.*
