# Supplementary material for: Intracardiac biopsy of cardiac tumors with echocardiographic guidance: Case report
Source: Front Cardiovasc Med. 2023 Apr 27;10:1103918. doi: 10.3389/fcvm.2023.1103918 (PMC10173306; doi:10.3389/fcvm.2023.1103918)
Supplement: Supplementary file 2 [file Table2.docx]

**Online Additional Videos:** **Intracardiac echocardiography (ICE) guided atrial biopsy**

Additional file 1: Video 1 for case1. Fluoroscopy image for placing the ICE and bioptome. ICE probe is placed across the tricuspid valve and positioned in the right ventricular inflow tract to image the ventricles in the long axis view. Through a long-deflectable sheath the bioptome is inserted and directed to the mass visualized by ICE. After checking the placement of the bioptome, a sample is taken for case1.

Additional file 2: Video 2 for case1. Cardiac mass biopsy guided by ICE. ICE visualizes a good contact between the bioptome and the mass for case1.

Additional file 3: Video 3 for case2. Fluoroscopy image for placing the ICE and bioptome for case2.

Additional file 4: Video 4 for case2. Cardiac mass biopsy guided by ICE for case2.

Additional file 5: Video 5 for case3. Fluoroscopy image for placing the ICE and bioptome for case3.

Additional file 6: Video 6 for case3. Cardiac mass biopsy guided by ICE for case3.
